# Recent advances regarding endoscopic biliary drainage for unresectable malignant hilar biliary obstruction

**DOI:** 10.1002/deo2.33

**Published:** 2021-09-07

**Authors:** Hironari Kato, Kazuyuki Matsumoto, Hiroyuki Okada

**Affiliations:** ^1^ Department of Gastroenterology and Hepatology Okayama University Graduate School of Medicine, Dentistry, and Pharmaceutical Sciences Okayama Japan

**Keywords:** biliary drainage, EUS‐BD, malignant hilar biliary obstruction, side‐by‐side, stent‐in‐stent

## Abstract

Biliary drainage for unresectable malignant hilar biliary obstruction (UMHBO) is still associated with a number of controversies to be resolved. The superiority of bilateral drainage in comparison to unilateral drainage has not been proven obviously yet. However, bilateral drainage is necessary to treat obstructive jaundice in some UMHBO patients, and this may be connected with preservation of the functional liver volume. The partial stent‐in‐stent (SIS) method and side‐by‐side (SBS) method developed as bilateral drainage methods. There is no significant difference in the technical or clinical success rates of the SIS and SBS methods. In addition, these methods are comparable in terms of adverse events, patency period, and survival period. On the other hand, reintervention for recurrent biliary obstruction (RBO) after the SBS method seems to be easier in comparison to cases with RBO after the SIS method; however, there is no remarkable difference in the clinical results of these procedures. Endoscopic ultrasound (EUS)‐guided biliary drainage also has become an option for patients with UMHBO. Left hepatic drainage using EUS‐guided hepaticogastrostomy (EUS‐HGS) has become common; however, few studies have reported the results of bridging drainage for the right lobe using the EUS‐HGS route or EUS‐guided hepaticojejunostomy. A few studies addressed the results of newly designed stents, such as the 6‐mm braided metal stent and inside stent. The development of various drainage methods and new devices is necessary for the further advancement of endoscopic biliary drainage for patients with UMHBO, further studies to evaluate those methods and devices are warranted.

**Abbreviations**
BTC: biliary tract cancer
EUS: endoscopic ultrasound
EUS‐BD: endoscopic ultrasound‐guided biliary drainage
FCSEMS: fully‐covered self‐expandable metal stent
HDS: hepaticoduodenostomy
HGS: hepaticogastrostomy
IS: inside stent
PCSEMS: partially‐covered self‐expandable metal stent
p‐RHD: posterior branch of right hepatic duct
PS: plastic stents
RBO: recurrent biliary obstruction
SBS: side‐by‐side
SEMS: self‐expandable metal stents
SIS: stent‐in‐stent
UMHBO: unresectable malignant hilar biliary obstruction

## INTRODUCTION

Recently, there have been remarkable advances in endoscopic drainage for biliary obstruction is remarkably advanced. Great advances have also been made regarding the methods and devices of endoscopic drainage for unresectable malignant hilar biliary obstruction (UMHBO) also made. However, in the management of UMHBO, there are various controversies that remain to be resolved because of the complex anatomical structure at the hilar portion.

When endoscopic biliary drainage for UMHBO is employed, the physician must decide whether to perform unilateral (single) stent placement or bilateral (multiple) stent placement. The selection of the number of stents is associated with the technical difficulty. Two types of stents, plastic stents (PSs) and self‐expandable metal stents (SEMSs), are available in various shapes, lengths, diameters, and structures. Various types of PSs and SEMSs are available, and the physician must choose an adequate stent according to the situation. When we employ bilateral biliary drainage, the partial stent‐in‐stent (SIS) method or side‐by‐side (SBS) method are mainly used for multiple SEMS placement. However, both methods are sometimes difficult, and the strategy of reintervention for SEMS obstruction is controversial. In addition to transpapillary endoscopic drainage, there have been remarkable advances in EUS‐guided biliary drainage (EUS‐BD) as an alternative drainage method for biliary obstruction. EUS‐BD has been reported as a rescue method for cases involving difficult multiple stent placement or difficult reintervention for UMHBO.

In this study, we reviewed recently published studies on endoscopic drainage for UMHBO and discussed the present status and future perspectives of endoscopic biliary drainage for UMHBO.

## UNILATERAL DRAINAGE VERSUS BILATERAL DRAINAGE

Bilateral drainage is thought to be more technically difficult than unilateral drainage. Unilateral drainage involves single SEMS placement in most cases, and it is natural that the success rate of single SEMS placement is almost 100%. On the other hand, it is difficult to achieve a success rate of 100% in bilateral drainage, which requires multiple SEMS placement. Aghaie Meybodi et al^1^ reviewed and analyzed 21 studies, which included 1292 UMHBO patients. They reported that the technical success in the unilateral drainage group (97%, 95% CI: 93%–98%) was significantly higher than that of the bilateral drainage group (89%, 95% CI: 84%–92%) (*p* = 0.0.003). It is difficult for bilateral drainage to show a technical advantage over unilateral drainage because of the technical process, although advances in endoscopy have reduced the technical differences between bilateral and unilateral drainage. However, due to advances in devices, such as SEMSs or guidewires and stent placement methods, most recent studies concluded that bilateral and unilateral drainage show statistically similar technical success rates.^2^, ^3^, ^4^ In addition, two meta‐analyses reported that the early and late complication rates of the two groups are similar.^1^, ^5^ The technical disadvantage of bilateral drainage has been reduced, and bilateral drainage has become common.

The advantage of bilateral drainage in comparison to unilateral drainage is thought to be the superior management of jaundice and cholangitis due to the drainage of a larger liver volume, which facilitates long stent patency and a long survival period. However, the clinical advantage of bilateral drainage has not been proven. Table 1 shows the results of a comparison between unilateral and bilateral drainage for UMHBO.^2^, ^3^, ^4^, ^6^, ^7^, ^8^ According to these results, bilateral drainage is not always necessary when initially performing drainage for patients with UMHBO. Takahashi et al^9^ concluded that drainage of ≥33% of the liver volume in patients with a preserved liver function and ≥50% in patients with an impaired liver function was correlated with the effective biliary drainage in UMHBO. Vienne et al^10^ reported the factors predicting the effectiveness of endoscopic drainage for UMHBO. The main factor associated with the effectiveness was a drained liver volume of >50%. If unilateral drainage meets these conditions, it is sufficient as the initial drainage for UMHBO.

**TABLE 1:** Comparison between unilateral and bilateral biliary drainage

	Number of patients		Technical success (% [*n*])			Clinical success (% [*n*])			Stent patency (months)			Survival period (months)		
Author, year	Unilateral	Bilateral	Unilateral	Bilateral	*p* value	Unilateral	Bilateral	*p* value	Unilateral	Bilateral	*p* value	Unilateral	Bilateral	*p* value
Naitoh, 2009	MS 17	MS 29	100 (17/17)	90 (26/29)		94 (16/17)	90 (25/26)		7.0	16.3	0.009	5.5	6.8	0.559
Iwano, 2011	MS 63	MS 19	95 (60/63)	90 (17/19)		NA	NA		4.4	4.2	0.322	5.7	6.1	0.4908
Mukai, 2013	PS 15	PS 15	100 (15/15)	100 (15/15)		100 (15/15)	100 (15/15)		3.4	3.7	0.746	NA	NA	
	MS 14	MS 16	100 (14/14)	100 (16/16)		100 (14/14)	100 (16/16)		12.1	9.8	0.347	NA	NA	
Lee, 2017	MS 66	MS 67	100 (66/66)	96 (64/67)	0.244	85 (56/66)	95 (61/64)	0.047	4.6	8.4	<0.01	5.9	9	0.053
Teng, 2019	MS 58	MS 52	93 (54/58)	90 (47/52)	0.864	96 (53/55)	98 (46/47)	1	6.1	6.6	0.999	6.3	6.6	0.867
Hakuta, 2021	ENBD36	ENBD39	100 (36/36)	95 (37/39)	0.49	57 (21/36)	56 (22/39)	0.99	NA	NA		NA	NA	
	MS 19	MS 25	100 (19/19)	100 (25/25)		NA	NA		11.3	4.3	0.11	9.7	7.9	0.79

Abbreviations: ENBD, endoscopic nasobiliary drainage; MS, metallic stent; NA, not available; PS, plastic stent.

The number of UMHBO patients who require bilateral drainage for initial drainage is an important issue. Mukai et al^4^ reported that approximately 50% of patients required bilateral drainage for the management of jaundice and cholangitis. Miura et al^11^ reported the results of preoperative biliary drainage for malignant hilar biliary obstruction, and 69 of 122 (56.7 %) patients required multiple biliary drainage by the time of the operation. Hakuta et al^6^ reported that 21/36 (57%) patients undergoing unilateral drainage by endoscopic nasobiliary drainage as initial drainage achieved clinical success. Although the evidence is insufficient, approximately half of UMHBO patients are expected to require bilateral drainage for initial drainage. In addition, the tumor gradually enlarges and occupies the drained area, and—as a result—the functional liver volume will decrease with the progression of the >50%. Considering these conditions, bilateral drainage is more reasonable than unilateral drainage for the preservation of the functional liver volume.

Uchida et al^12^ reported the effectiveness of multi‐sectional biliary drainage in patients treated with chemotherapeutic agents for unresectable biliary tract cancer (BTC). The patients were divided into a group with the placement of three of four SEMSs (3‐ or 4‐branched group) and a group with the placement of one or two SEMSs (one‐ or two‐branched group). Although the patency and the survival period did not differ between the two groups, the patency and survival period in the three‐ or four‐branched group were significantly longer in comparison to the those in the one‐ or two‐branched group in patients with disease control by chemotherapy. They argued that multi‐sectional biliary drainage prevented biliary infection and maintained the functional liver volume, which enabled the patients in the disease control group to continue to receive effective chemotherapy; thus, patients in the four‐ or three‐branched group achieved longer stent patency and survival.

## METHODS FOR MULTIPLE SEMS PLACEMENT

The partial SIS method and SBS method are well known as methods for multiple SEMS placement. Both methods are classical, and approximately 20 years have passed since they were reported.^13^, ^14^ In the SIS method, placement of the second SEMS is associated with a high degree of difficulty. At the time of placement of the second SEMS, it is often difficult to advance the guidewire or to deliver the SEMS through the stent mesh and biliary obstruction. However, various improvements of stent mesh have made SIS easier.^15^, ^16^ When the SBS method was started, the simultaneous insertion of two SEMS delivery systems into the working channel of the endoscope was impossible because of the thickness of the SEMS delivery system and the small working channel of the endoscope. The insertion of a second SEMS beside the first SEMS after placement was often difficult. However, a thinner SEMS delivery system and larger endoscope working channel made the SBS method easier.^17^ Hence, these two methods are mainly used for multiple SEMS placement. Several studies have compared the SIS and SBS methods (Table 2).^18^, ^19^, ^20^, ^21^ Due to these advances in methods and devices, it seems that there are no obvious differences between SIS and SBS as the initial drainage method.

**TABLE 2:** Comparison between the SIS and SBS methods

	Number of patients		Technical success (% [*n*])			Clinical success (% [*n*])			Early AE, Late AE (%)			Stent patency (months)			Survival period (months)		
Author, year	SIS	SBS	SIS	SBS	*p* value	SIS	SBS	*p* value	SIS	SBS	p value	SIS	SBS	*p* value	SIS	SBS	*p* value
Kim, 2012	22	19	100 (22/22)	100 (19/19)		82 (18/22)	79 (15/19)	1	32 37	23 50	0.73 0.53	4.5	3.9	0.77	7.5	4.9	0.77
Naitoh, 2012	24	28	100 (24/24)	89 (17/19)	0.15	100 (24/24)	96 (24/25)	0.51	4 8	11 32	0.37 0.074	3.5	5.2	0.39	5.3	6.6	0.95
Lee, 2019	34	35	100 (34/34)	91 (32/35)	0.081	94 (32/34)	91 (29/32)	0.67	12 18	11 23	0.97 0.59	8.4	8.7	0.87	7	7.4	0.2
Ishigaki, 2020	40	24	100 (40/40)	96 (23/24)	0.99	93 (37/40)	96 (23/24)	0.99	23 10	46 12	0.09 0.99	5.6	6.8	0.67	7.9	12.7	0.07

Abbreviations: AE, adverse events; SBS, side‐by‐side; SIS, stent‐in‐stent.

**FIGURE 1:**
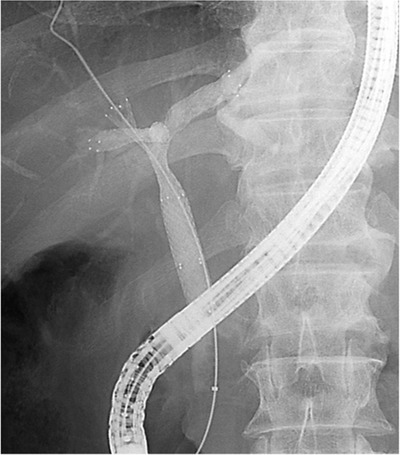
Three SEMS placement with the SIS method

**FIGURE 2:**
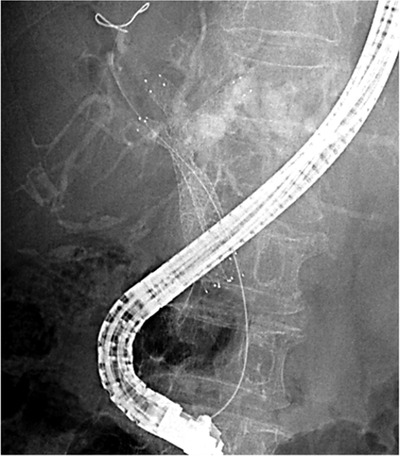
Three SEMS placement with a combination of the SIS and SBS methods

Along with the methods and devices, several studies have addressed three SEMS placement. The entire liver is divided into three segments; the left lobe, the anterior segment of the right lobe, and the posterior segment of the right lobe, and the three SEMS are placed in the three segments (Figure 1). Kawamoto et al^22^ reported that three SEMS placement with the SIS method was employed in nine cases. During the follow‐up period (mean 5.0 months, range 2.7–16.4 months), three patients (33%) developed SEMS obstruction. The other six patients did not develop SEMS obstruction during the follow‐up period (mean 11 months, range 4.7–16.4 months). The most difficult part of three SEMS placement with the SIS method was the insertion of the guidewire and stent delivery system through the two stent meshes and biliary obstruction, and the operator must maneuver the guidewire and various devices (e.g., several types of guidewires and dilators). Koshitani et al^23^ reported that successful three SEMSs placement was achieved with the combination of SIS and SBS methods. Two SEMSs with 6‐Fr delivery system were placed with the SBS method into the left hepatic duct and the posterior branch of the right hepatic duct (p‐RHD). Then, another SEMS was placed by the SIS method for the SEMS placed in the p‐RHD into the anterior branch of the right hepatic duct (Figure 2). The advantage of this method is that it is not necessary to negotiate two stent meshes or biliary obstruction, which is one of the causes of placement failure. They reported that technical and clinical success was achieved in 11 cases, including one case involving the placement of four SEMSs, and the median stent patency was 5 months during a mean follow‐up period of 6.1 months (range: 1.2–18.6).^24^ Maruki et al^25^ reported the results of 17 cases in which three SEMSs placement was performed with a combination method using SEMS with a 5.4‐Fr or 5.7‐Fr delivery system. The technical success rate was 82% (14/17), and the median procedure time was 54 min. Two patients (12%) developed cholecystitis as early adverse events, and one (6%) developed liver abscess as a late adverse event. The time to RBO among patients with successful initial trisegmental drainage was 189 days (95% confidence interval, 124–254).

## REINTERVENTION FOR STENT OBSTRUCTION IN PATIENTS WITH MULTIPLE SEMS PLACEMENT

Most cases of UMHBO are caused by BTC, and recent effective chemotherapeutic regimens with cancer drugs, molecular targeted drugs, and immune checkpoint inhibitors have improved the prognosis of the patients with BTC. The overall survival of BTC patients treated with various chemotherapies is approximately one year or more. Thus, initial stent occlusion often occurs in these patients, and adequate reintervention is important for continuous chemotherapy, especially in patients with bilateral SEMS placement.

The results of reintervention for patients with RBO after initial bilateral stent placement are shown in Table 3.^19^, ^26^, ^27^, ^28^, ^29^, ^30^, ^31^, ^32^, ^33^, ^34^, ^35^, ^36^ According to these reports, the technical success rate is 67%–100%, and the rate is thought to be affected by the definition of technical success (successful unilateral stent placement or successful bilateral stent placement); thus, the comparison of technical success rates among these reports is difficult. The clinical success rate was 52%–100%, and most studies reported clinical success in most cases in which technical success was achieved. On the other hand, the rate of bilateral stent placement was 49%–100%, which was lower than the clinical success rate in most studies. This result shows that bilateral stent placement is not always necessary for clinical improvement in patients with RBO after initial bilateral SEMS placement, although continuous bilateral drainage is thought to be desirable due to the preservation of the functional liver volume. Regarding stents, PSs are more frequently used for reintervention than SEMSs. PS placement is expected to be more feasible than SEMS placement for reintervention in cases of bilateral SEMS placement, especially with the SIS method.

**TABLE 3:** Comparison of reintervention with the SIS and SBS methods

Author	Placement method	Number of patients	Technical success (% [*n*])	Clinical success (% [*n*])	Multiple drainage at reintervention (% [*n*])	Stent
Naitoh, 2012	SIS	10	90 (9/10)	NA	NA	PS 6, SEMS 2
	SBS	5	100 (5/5)	NA	NA	PS 1, SEMS 4
Lee, 2013	SBS	18	67 (12/18)	NA	50 (6/12)	PS 8, SEMS 4
Fujii, 2013	SIS	30	100 (30/30)	100 (30/30)	67 (20/30)	PS 21, SEMS 4, Cleaning 5
Lee, 2013	SIS	24	83 (20/24)	95 (19/20)	83 (20/24)	PS 9, SEMS 11
Law, 2013	SIS	3	100 (3/3)	NA	100 (3/3)	PS 3, SEMS 5, Cleanign 1
	SBS	8	75 (6/8)	NA	75 (6/8)	
Inoue, 2016	SIS	24	92 (48/52)	90 (43/48)	61 (29/52)	PS 33, SEMS 15
	SBS	28				
Hong, 2017	SIS	12	83 (10/12)	80 (8/10)	50 (6/12)	PS 5, SEMS 3, PS+SEMS 2
Tomoda, 2017	SIS	33	82 (27/33)	100 (27/27)	82 (27/33)	PS 33
Son, 2018	SIS	38	76 (29/38)	52 (15/29)	21 (8/38)	PS 19, SEMS 6, Cleaning 4
Okuno, 2019	SIS	31	81 (25/31)	100 (25/25)	NA	PS 14, SEMS 4, ENBD 7
Inoue, 2020	SBS	67	79 (53/67)	96 (51/53)	79 (50/67)	PS 38, SEMS 12, Cleaning 3
Lee, 2020	SIS	48	96 (46/48)	72 (33/46)	49 (27/55)	PS 28, SEMS 27
	SBS	7	71 (5/7)	100 (5/5)		

Abbreviations: ENBD, endoscopic nasobiliary drainage; NA, not available; PS, plastic stent; SBS, side‐by‐side; SEMS, self‐expandable metal stent; SIS, stent‐in‐stent.

**FIGURE 3:**
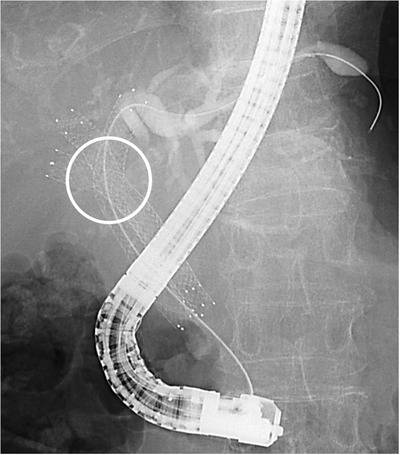
SEMSs placed with the SBS method cross and partially overlap at the hilar portion (round)

Theoretically, it is not necessary to advance the guidewire and stent delivery system through the stent mesh and obstruction when reintervention is performed for patients with bilateral drainage by the SBS method; this is one of advantages of the SBS method over the SIS method. Thus, reintervention for patients with bilateral SEMS placement by the SBS method is considered to be easier to apply than that by the SIS method. However, there is no difference in the technical success rates of these procedures. Inoue et al^32^ analyzed the factors associated with technical failure of reintervention for RBO after initial bilateral SEMS placement and reported that the method of bilateral SEMS placement was not significantly associated with the technical failure of reintervention. Laser‐cut SEMSs, the delivery system of which is thinner in comparison to braided SEMS, are usually used in the SBS method. Two laser‐cut SEMSs placed by the SBS method sometimes cross and partially overlap at the bifurcation of the right and left hepatic ducts because of the structure of the laser‐cut SEMSs (Figure 3). In this situation, the insertion of the guidewire and stent delivery system into the obstructed SEMS after the SBS method becomes complex, similar to that after the SIS method.

## THE FEASIBILITY AND EFFECTIVENESS OF EUS‐BD FOR UMHBO

**FIGURE 4:**
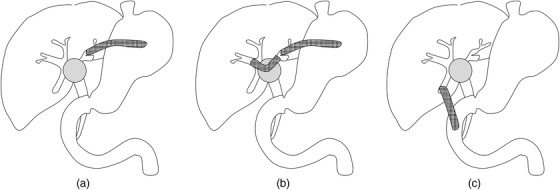
(a) Left hepatic drainage with EUS‐HGS. (b) Right hepatic drainage with bridging SEMS placement through the EUS‐HGS route. (c) Right hepatic drainage with EUS‐HDS

EUS‐BD has become a biliary drainage option, in addition to the existing options of endoscopic drainage and percutaneous drainage. EUS‐BD for UMHBO is applied for initial drainage and rescue drainage (e.g., difficult drainage through the papilla or reintervention after initial drainage through the papilla), and several studies have reported results of this procedure. Three methods of EUS‐BD for UMHBO have been reported (Figure 4): left hepatic drainage with EUS‐guided hepaticogastrostomy (EUS‐HGS),^37^, ^38^ and right hepatic drainage with bridging SEMS placement through the EUS‐HGS route,^39^ and right hepatic drainage with EUS‐guided hepaticoduodenostomy (EUS‐HDS).^40^, ^41^, ^42^ Although left hepatic drainage with EUS‐HGS is becoming common, few studies have addressed right hepatic drainage with EUS‐BD. Moryoussef et al^43^ reported that bridging SEMS placement was successful in three of six procedures (50%) for five patients, and that the clinical success rate was 60% (3/5). Caillol et al^44^ reported that all 12 patients (100%) achieved successful placement of bridging SEMS, and that clinical success was achieved in 10 patients (83%). Ogura et al^45^ reported that bridging SEMS placement was employed in nine patients, and that technical success was achieved in all nine patients. Bridging SEMS placement is applied to initial drainage in these all studies. This procedure is expected to be more difficult in cases with reintervention after SEMS placement through the papilla, especially in the case of bilateral SEMS placement. Similarly, few studies have addressed right hepatic drainage with EUS‐HDS. Park et al^42^ reported that EUS‐HDS was employed for three patients with no adverse events. Ogura et al reported that EUS‐HDS was employed with no adverse events for four and two patients in different studies.^45^, ^46^ The candidates of these two methods are limited. Bridging insertion of the guidewire and stent delivery system is sometimes difficult due to the sharp angle of the hilar portion. Successful detection and the approach to the posterior branch of the right hepatic duct cannot be always achieved. More studies with a large number of patients are warranted to evaluate the feasibility and usefulness of right hepatic drainage with bridging SEMS placement through the left HGS route and EUS‐HDS.

## OTHER STENTS FOR BILATERAL STENT PLACEMENT

Recently, a few studies have addressed the efficacy and feasibility of the 6‐mm braided SEMS. Because of the structure, bilateral placement of braided SEMSs with the SBS method can avoid overlapping at the hilar portion, which results in easy reintervention. In addition, the 6‐mm size is more physiological when multiple SEMSs are placed with the SBS method.^47^ Inoue et al^48^ reported the results of bilateral drainage using a 6‐mm threaded fully‐covered SEMS (FCSEMS) placed above the papilla with the SBS method. The technical success rate was 94% (16/17), and the median time to RBO was 7 months. The FCSEMS could be removed in all patients, and the reintervention success rate was 100%. Kitamura et al^49^ reported results of 17 patients undergoing 6‐mm partially‐covered SEMS (PCSEMS) placement with the SBS method. The technical success rate was 100%, and the median time to RBO was 2.6 months. The PCSEMS could be removed in six of 12 patients with RBO, and a new SEMS could be placed with the SBS method in six patients with successful stent removal. On the other hand, a new SEMS could be placed with the SIS method in six patients without successful stent removal. Gao et al^50^ reported the results of 45 patients undergoing 6‐mm uncovered SEMS placement. The technical success rate was 100%. The median time to RBO was 8.7 months, and the success rate of reintervention was 100%. Irrespective of the small number of studies about 6‐mm braided SEMSs, the results are preferable, and more studies are warranted.

The long patency of the biliary stent is important for maintaining the quality of life of patients with obstructive jaundice due to incurable malignant disease. Although PS placement is easier than SEMS placement, especially in cases with bilateral stent placement, SEMSs have been used more frequently than PSs due to their long patency. However, as previously mentioned, bilateral SEMS placement is associated with various controversies. Several studies have reported the effectiveness of the “inside stent (IS).”^51^, ^52^, ^53^, ^54^ An IS is a threaded PS, the proximal end of which is above the papilla after placement. The proximal end of the usual PS is in the duodenum after placement, which causes the reflux of duodenal juice into the stent and bile duct. The reflux of duodenal juice is thought to be the cause of biofilm formation and bacterial adherence to the wall of the stent, which is connected with stent obstruction. ISs are thought to have long patency because avoid the reflux of duodenal juice into the stent and the bile duct is avoided after stent placement. Inatomi et al^51^ reported the results of comparison among conventional PSs, SEMSs, and ISs for patients with UMHBO. ISs had a significantly longer patency period in comparison to conventional PSs (142 vs. 32 days, *p* = 0.04), and there was no difference in the patency period between ISs and SEMSs (142 vs. 150 days, *p* = 0.83). Kanno et al^54^ reported the results of endoscopic drainage using an SEMS or IS for the patients with UMHBO, including unilateral drainage. The patency period of the IS group was significantly longer than that of the SEMS group (IS vs. SEMS: 561 days vs. 209 days, *p* = 0.008). More studies are warranted to conclude whether the patency period of the IS is comparable or superior to the SEMS. The advantage of PSs, including ISs, is their removability, and the increase of conversion surgery or effective chemotherapy will bring opportunities for PS placement.

## CONCLUSION

We described the current status of endoscopic biliary drainage in patients with UMHBO and the associated controversies. At present, a complete cure is difficult to achieve for all MHBO patients, and advances in biliary drainage are therefore essential. The development of various drainage methods and new devices including SEMSs and PSs are necessary for the further advancement of endoscopic biliary drainage for the patients with UMHBO. In addition, numerous studies should be performed to evaluate these methods and devices.

## CONFLICT OF INTEREST

The authors declare that there is no conflict of interest that could be perceived as prejudicing the impartiality of the research reported.

## FUNDING INFORMATION

None.
